# A Novel Model Based on CXCL8-Derived Radiomics for Prognosis Prediction in Colorectal Cancer

**DOI:** 10.3389/fonc.2020.575422

**Published:** 2020-10-14

**Authors:** Yanpeng Chu, Jie Li, Zhaoping Zeng, Bin Huang, Jiaojiao Zhao, Qin Liu, Huaping Wu, Jiangping Fu, Yin Zhang, Yefan Zhang, Jianqiang Cai, Fanxin Zeng

**Affiliations:** ^1^Department of Clinical Research Center, Dazhou Central Hospital, Dazhou, China; ^2^Department of Cardiology, Peking University First Hospital, Beijing, China; ^3^Department of Gastrointestinal Surgery, Nanchong Central Hospital, Nanchong, China; ^4^Department of Oncology, Dazhou Central Hospital, Dazhou, China; ^5^Department of Hepatobiliary Surgery, National Cancer Center/Cancer Hospital, Chinese Academy of Medical Sciences and Peking Union Medical College, Beijing, China; ^6^School of Medicine, Sichuan University of Arts and Science, Dazhou, China

**Keywords:** colorectal cancer, CXCL8, transcriptomics, radiomics, prognosis

## Abstract

**Introduction:** Prognosis prediction is essential to improve therapeutic strategies and to achieve better clinical outcomes in colorectal cancer (CRC) patients. Radiomics based on high-throughput mining of quantitative medical imaging is an emerging field in recent years. However, the relationship among prognosis, radiomics features, and gene expression remains unknown.

**Methods:** We retrospectively analyzed 141 patients (from study 1) diagnosed with CRC from February 2018 to October 2019 and randomly divided them into training (*N* = 99) and testing (*N* = 42) cohorts. Radiomics features in venous phase image were extracted from preoperative computed tomography (CT) images. Gene expression was detected by RNA-sequencing on tumor tissues. The least absolute shrinkage and selection operator (LASSO) regression model was used for selecting imaging features and building the radiomics model. A total of 45 CRC patients (study 2) with immunohistochemical (IHC) staining of *CXCL8* diagnosed with CRC from January 2014 to October 2018 were included in the independent testing cohort. A clinical model was validated for prognosis prediction in prognostic testing cohort (163 CRC patients from 2014 to 2018, study 3). We performed a combined radiomics model that was composed of radiomics score, tumor stage, and *CXCL8*-derived radiomics model to make comparison with the clinical model.

**Results:** In our study, we identified the *CXCL8* as a hub gene in affecting prognosis, which is mainly through regulating cytokine–cytokine receptor interaction and neutrophil migration pathway. The radiomics model incorporated 12 radiomics features screened by LASSO according to *CXCL8* expression in the training cohort and showed good performance in testing and IHC testing cohorts. Finally, the *CXCL8*-derived radiomics model combined with tumor stage performed high ability in predicting the prognosis of CRC patients in the prognostic testing cohort, with an area under the curve (AUC) of 0.774 [95% confidence interval (CI): 0.674–0.874]. Kaplan–Meier analysis of the overall survival probability in CRC patients stratified by combined model revealed that high-risk patients have a poor prognosis compared with low-risk patients (Log-rank *P* < 0.0001).

**Conclusion:** We demonstrated that the radiomics model reflected by *CXCL8* combined with tumor stage information is a reliable approach to predict the prognosis in CRC patients and has a potential ability in assisting clinical decision-making.

## Introduction

Colorectal cancer (CRC) is a common gastrointestinal tract malignancy. The incidence of CRC has a clear upward trend (1, 2) with the changes in human diet and lifestyle. In 2018, there were more than 1.8 million new cases of CRC worldwide, and accounting for about 850,000 deaths per year (3). Although great improvements have been achieved due to effective screening tools, refined surgical techniques, and molecular target drugs, the prognosis of CRC patients was not yet satisfactory. The 5-year survival rate of CRC patients with stage I or II cancer is >75%. However, more than 20% of patients have already progressed to a distant stage at the first diagnosis, and the 5-year survival rate was only 14% (4). Therefore, early prognosis prediction and subsequent individual therapy strategies are greatly beneficial for CRC patients.

*CXCL8*, also known as interleukin-8, is a member of the chemokine family and is involved in inflammation and immune response (5). Previous studies have revealed an abnormal high expression of *CXCL8* in malignant tumors, which is mainly involved in tumor cell growth, apoptosis, invasion, and migration (6–12). Large-scale cohort studies have suggested that elevated plasma or serum *CXCL8* was associated with worse prognosis in melanoma, metastatic urothelial carcinoma, and renal cell carcinoma patients (13, 14). In terms of CRC, some studies have demonstrated that a high level of *CXCL8* could promote poor overall and disease-free survival. *In vitro* experiments implicated that *CXCL8* modulated CRC cells anoikis or induced the epithelial–mesenchymal transition to result in poor outcomes (8, 15). However, the underlying mechanistic connections among *CXCL8*, phenotypes, and CRC prognosis are extensively unknown.

Radiomics is an emerging and effective method for quantitative analysis based on high-throughput features of medical imaging, which mainly involves four steps: image acquisition, imaging segmentation, feature extraction and quantification, and feature selection and modeling (16). Increasing evidence presents the advantages of radiomics as a noninvasive approach in early diagnosis, prognosis prediction, and curative effect evaluation of tumors (17–19). In CRC patients, studies have demonstrated the successful application of radiomics in the prediction of lymph node metastasis and of outcomes and evaluation of sensitivity to drug therapy (19–21). Many studies focused on radiomics features or combined clinical factors and gene expression with radiomics in CRC; however, to our knowledge, the potential associations between radiomics, gene expression, and CRC remain to be fully elaborated.

Therefore, in our study, we aimed to explore the potential connections between *CXCL8* expression and imaging features and further develop and validate a radiomics model derived from *CXCL8* expression for individual preoperative prediction of prognosis in CRC patients.

## Materials and Methods

### Patients

This study included three studies (cohorts) with a total of 355 patients who were pathologically diagnosed with CRC at Dazhou Central Hospital. Study 1 consisted of 147 patients (87 males and 60 females, mean age: 60.73 years), who underwent surgery from February 2018 to October 2019. CRC tissues and their adjacent normal tissues were collected and stored immediately in liquid nitrogen for RNA sequencing (RNA-seq) after radical resection. Among them, the RNA-seq data of 95 paired tissues from 1 year were used for bioinformatic analysis. Study 2 included 45 patients (27 males and 18 females, mean age: 63.28 years) with paraffin-embedded tumor tissues at the Department of Pathology from 2014 to 2018. Study 3 contained 163 patients (95 males and 68 females, mean age: 63.94 years) from 2014 to 2018. The clinical stage of tumors was classified according to tumor–node–metastasis (TNM) staging system [American Joint Committee on Cancer (AJCC) 8th edition staging system]. All patients' clinical information was obtained by electronic medical record. Detailed information of these patients is shown in Table 1 and Supplementary Table 1. Patients in different studies have no significant difference in age, sex distribution, tumor stage, and tumor site proportion.

**Table 1:** Characteristics of patients in three studies.

**Characteristic**	**Study 1 (*n* = 147)**	**Study 2 (*n* = 45)**	**Study 3 (*n* = 163)**	***P***
Age (mean ± SD)	60.73 ±11.99	63.28 ± 10.38	63.94 ± 10.02	0.082
Sex, No. (%)				0.974
Male	87 (58.95)	27 (60.00)	95 (58.28)	
Female	60 (41.05)	18 (40.00)	68 (41.72)	
Tumor stage, No. (%)				0.202
0	4 (2.72)	0 (0.00)	0 (0.00)	
I	38 (25.85)	14 (31.11)	39 (23.93)	
II	40 (27.21)	10 (22.22)	59 (36.20)	
III	53 (36.05)	16 (35.56)	55 (33.74)	
IV	12 (8.16)	5 (11.11)	10 (6.14)	
Tumor sites, No. (%)				0.984
Rectum	100 (68.03)	31 (68.89)	109 (66.87)	
Right colon	24 (16.33)	7 (15.56)	23 (14.11)	
Left colon	21 (14.29)	6 (13.33)	28 (17.18)	
Multiple tumors	2 (1.36)	1 (2.22)	3 (1.84)	

*The P-value of age: Kruskal-Wallis rank test. The P value of sex, tumor stage, tumor sites: chi-square test*.

To explore the relationship between *CXCL8* levels and overall survival in patients with tumors, data from two phase 3 clinical studies (CheckMate 067 and CheckMate 025) were downloaded (14). CheckMate 067 included 887 patients with melanoma treated with nivolumab or ipilimumab or nivolumab plus ipilimumab. CheckMate 025 included 392 patients with renal cell carcinoma treated with nivolumab.

**Figure 1:**
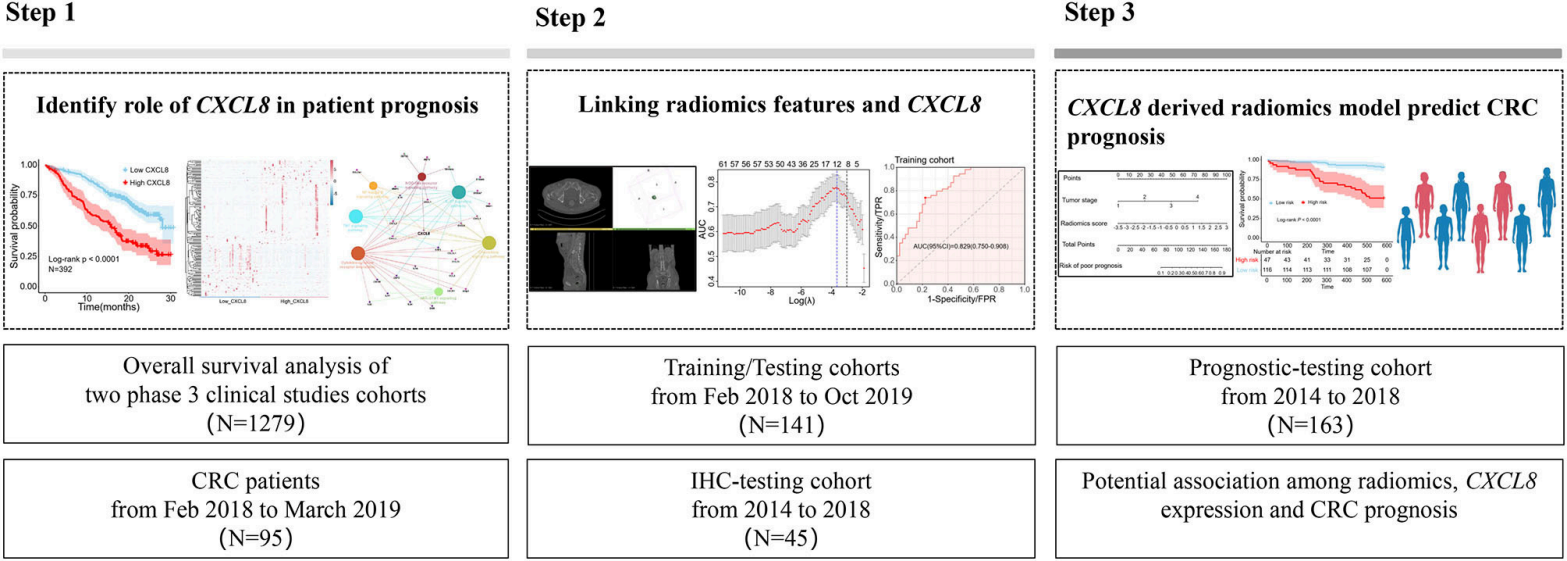
Workflow of the study. IHC, immunohistochemistry; CRC, colorectal cancer.

The study was approved by the medical ethics review committee of Dazhou Central Hospital (IRB00000003-17003). Written informed consent was obtained from patients in study 1 and study 2. The medical ethics review board waived the need for informed consent for study 3. The workflow of this study is presented in Figure 1.

### RNA-Sequencing

Total RNA of each sample was extracted by Trizol (TaKaRa Biomedical Technology, Beijing, China) according to the kit instructions. NanoPhotometer spectrophotometer and Agilent 2100 bioanalyzer were used to measure RNA purity and integrity, respectively. The mRNA was further enriched with Oligo (dT) magnetic beads and randomly interrupted with a bivalent cation in NEB Fragmentation Buffer. The library was constructed according to the NEB library construction method. After the library construction was completed, Qubit 2.0 Fluorometer was used for preliminary quantification. Subsequently, the insert size of the library was detected by Agilent 2100 bioanalyzer to ensure the quality of library. Qualified libraries were sequenced by Illumina with the sequencing strategy PE150. The original data file obtained by high-throughput sequencing was transformed into the raw data by CASAVA Base Calling analysis. Clean data for subsequent analysis were obtained by filtering the raw data, checking the sequencing error rate, and checking the GC content distribution. *CXCL8* expression levels in 147 patients of study 1 (141 had CT data, 6 had no CT data) were reflected by relative TPM value (TPM value in CRC tumors/TPM value in adjacent normal tissues) in transcriptome analysis. Patients with relative value above 10 were included in the high expression group and below 10 were included in the low expression group.

### Immunohistochemistry

Each paraffin-embedded tumor tissue was cut into 4-μm-thick sections by pathological sectioning machine (RM2245, Leica, Germany) and placed on a glass slide. The pathological sections were placed in a Water Bath-Slide Drier (PHY-III, China) and baked at 65°C for 2 h, and then after cooling, dewaxed twice with xylene for 20 min each time. The slices treated with different gradient concentrations of alcohol were repaired with EDTA repair solution. Then, 3% peroxidase blocker and blocking solution were added successively. *CXCL8* antibody (1:100, Affinity, USA) was used to analyze the sections. The results were collected by microscope (BA600-4, Motic, China) after sealing. The immunohistochemistry (IHC) score was calculated as follows: total score = intensity score × percentage score. Intensity score was based on staining intensities and mainly classified into four levels: negative (0), weak (1), moderate (2), and strong (3). Percentage score was based on the percentages of positive cells in staining and classified into five levels: <5% (0), 5–25% (1), 25–50% (2), 50–75% (3), and >75% (4). The IHC score ranging from 0 to 4 was defined as low *CXCL8* group and ranging from 5 to 12 was defined as high *CXCL8* group.

### CT Image Acquisition and Radiomics Signature Extraction

All patients underwent abdominal CT enhancement scan using SOMATOM Definition AS 64-slice CT (SIEMENS, Germany) before surgery. The parameters were as follows: 100 KV, CARE Dose4D, 0.5 s rotation time; detector collimation: 128 × 0.6 mm, field of view: 380 × 380 mm, matrix: 512 × 512. After the conventional CT scan, 60–80 ml of ioversol contrast agent (320 mgI/ml) was injected with a speed of 2–3 ml/s and a high-pressure syringe (Ulrich, Germany), and then injected normal saline (40 ml), arterial scan after 23 s, venous scan after 60 s, and delayed scan after 120 s. Prolong the delayed scan time appropriately according to the lesion. Contrast-enhanced CT reconstruction, the reconstruction thickness was 1 mm. DICOM data were retrieved from INFINITT Healthcare Co. Ltd. (Korea). The preoperative CT images of 349 patients enrolled in radiomics analysis from 2014 to 2019 were obtained and saved in DICOM format (Supplementary Figure 1). Among them, 141 patients with CT data were randomly divided into training cohort and testing cohort, 45 patients with IHC staining *CXCL8* were grouped as the IHC testing cohort, 163 patients with prognostic information were grouped as the prognostic testing cohort. Two professional observers used the 3D Slicer software to delineate the tumor tissue under the guidance of a clinical imaging specialist for building a 3D tumor tissue (22). Finally, a total of 854 radiomics eigenvalue results derived from each patient's CT images were collected for analysis.

### Statistical Analysis

Continuous data were presented as the mean ± standard deviation (SD), and Kruskal–Wallis rank test was performed to compare the difference among the three studies. Chi-square test was used to explore the difference in sex, tumor stage, and tumor site distribution. Differentially expressed genes (DEGs) were analyzed using the DESeq2 package in R software (Version: 3.6.3). |log2 Fold change| ≥ 1 and *p*-adjust < 0.05 were chosen as the cutoff value for identifying DEGs. Gene Ontology (GO) and Kyoto Encyclopedia of Genes and Genomes (KEGG) pathway analysis were performed by Cytoscape (Version: 3.6.1) plug-in ClueGO and CluePedia, with adj_*P*-value (adjusted by Bonferroni) < 0.05. Mann–Whitney *U* test and Least absolute shrinkage and selection operator (LASSO) algorithm were performed for potential radiomics feature selection. The LASSO regression model was often adopted for feature selection in high-dimensional data (23). An appropriate tuning parameter selection (λ) with 8-fold cross validation was calculated in the LASSO, where radiomics features with LASSO coefficient unequal to zero were selected. Multivariable logistic regression analysis was used to establish the combined model. Kolmogorov–Smirnov (KS) curve and receiver operating characteristic (ROC) curve were conducted to evaluate the performance of the models. Figures were created using GraphPad Prism 8, Cytoscape, and R software. Rpackages “sampling” (version 2.8), “dplyr” (version 0.8.3), “pROC” (version 1.15.3), “purrr” (version 0.3.3), “tidyr” (version 1.0.0), “ggplot2” (version 3.2.1), “stringr” (version 1.4.0), “rmda” (version 1.6), “glmnet” (version 3.0-2), “survival” (version 3.1-8), “rms” (version 0.2.8.1), “givitiR” (version 1.3), and “data.table” (version 1.12.8) were used. *P*-value < 0.05 was considered statistically significant.

## Results

### Identify the Role of *CXCL8* in Colorectal Cancer

**Figure 2:**
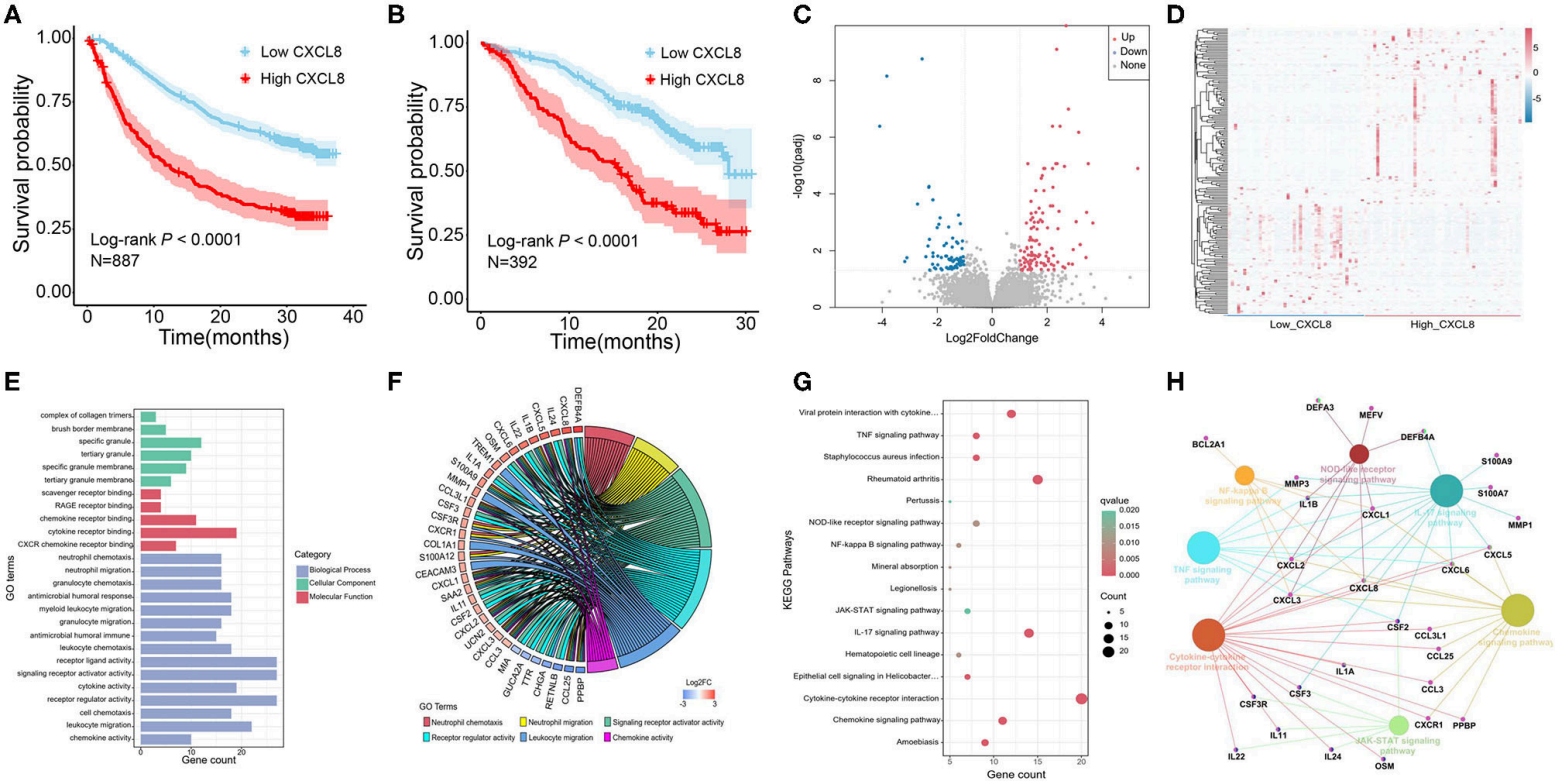
The effect of *CXCL8* expression level on the prognosis of colorectal cancer (CRC) patients. **(A)** Overall survival of melanoma patients with low *CXCL8* and high *CXCL8* expression (CheckMate 067). **(B)** Overall survival of renal cell carcinoma patients with low *CXCL8* and high *CXCL8* expression (CheckMate 025). **(C)** Volcano plot of differentially expressed genes (DEGs) between low *CXCL8* and high *CXCL8* groups. **(D)** Heatmap analysis of DEGs between low *CXCL8* and high *CXCL8* groups. **(E,F)** Gene Ontology (GO) analysis of DEGs. **(G,H)** Kyoto Encyclopedia of Genes and Genomes (KEGG) analysis of DEGs.

We analyzed the relationship between *CXCL8* expression and the prognosis in two phase 3 clinical studies [CheckMate 067 (melanoma); CheckMate 025 (renal cell carcinoma)] (14). As shown in Figures 2A,B, patients with low *CXCL8* levels had a better prognosis (*N* = 887, Log-rank *p* < 0.0001; *N* = 392, Log-rank *p* < 0.0001). In CRC patients, the study has reported that high *CXCL8* levels in tumors were associated with poor prognosis (8). Furthermore, RNA-seq with 95 CRC tissues in our study was performed to explore the underlying mechanisms of different *CXCL8* expressed levels in CRC. Compared with the low *CXCL8* expressed group, 185 DEGs were identified in the high *CXCL8* expressed group, with 112 genes upregulated and 73 genes downregulated (Figures 2C,D).

GO and KEGG pathway enrichment analyses were performed on 185 DEGs to illustrate their biological functions in CRC. The GO analysis totally revealed 55 significantly enriched GO terms, mainly including biological process, cellular component, and molecular function analysis. The top 15 GO terms of biological process and all GO terms of both cellular component and molecular function are displayed in Figure 2E. In biological process analysis, neutrophil chemotaxis, neutrophil migration, signaling receptor activator activity, receptor regulator activity, leukocyte migration, and chemokine activity were the terms most related to *CXCL8* (Figure 2F). There were 16 significant KEGG enrichment pathways, in which chemokine signaling pathway, cytokine–cytokine receptor interaction, nuclear factor (NF)-κB signaling pathway, NOD-like receptor signaling pathway, and IL-17 signaling pathway were highly associated with *CXCL8* (Figures 2G,H). These results suggested that *CXCL8* may affect CRC prognosis through regulating the above significant GO terms and KEGG pathways.

### Link of Radiomics Features and *CXCL8* Expression

**Figure 3:**
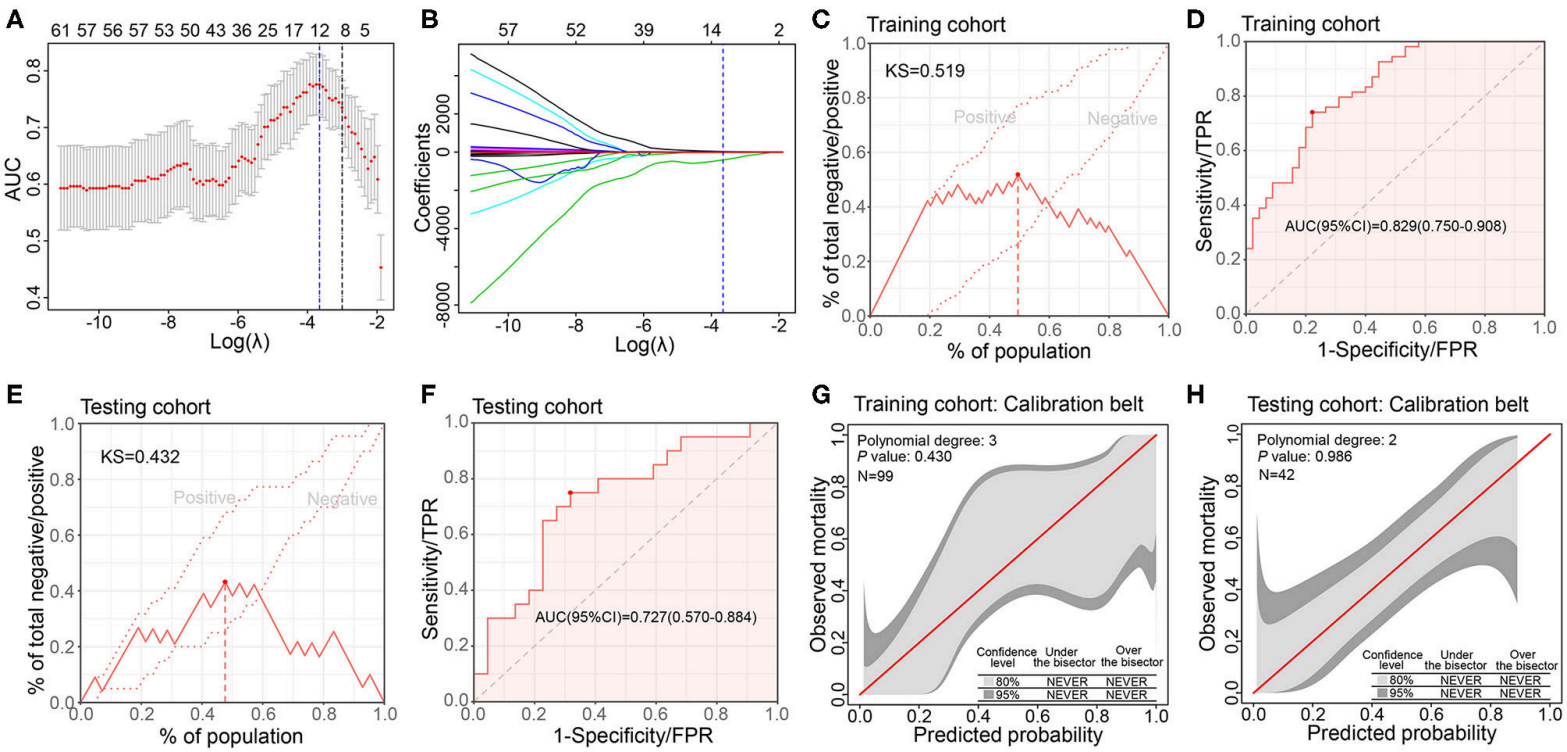
Development, validation, and performance of radiomics model for assessing *CXCL8* expression in the training and testing cohorts. **(A)** Tuning parameter selection (λ) with 8-fold cross validation in the Least absolute shrinkage and selection operator (LASSO) model. The area under the curve (AUC) of receiver operating characteristic (ROC) curve is plotted against log (λ). The dotted vertical lines represent the optimal values by minimum criteria and the 1 standard error of the minimum (1-SE) criteria. **(B)** LASSO coefficient profiles of the 87 radiomics features. **(C,D)** Kolmogorov–Smirnov (KS) curve and ROC curve of radiomics model in assessing *CXCL8* expression in the training cohort (*N* = 99). **(E,F)** KS curve and ROC curve of radiomics model in assessing *CXCL8* expression in the testing cohort (*N* = 42). **(G,H)** Calibration belt of the radiomics model for *CXCL8* expression assessment in the training and testing cohorts.

To link the *CXCL8* expression and radiomics features, we developed and validated a model based on radiomics features to evaluate the *CXCL8* expression (detected by RNA-seq) in 141 CRC patients. Of the extracted radiomics features firstly screened by Mann–Whitney *U* test (*P* < 0.01), 12 of 87 features were finally selected as potential predictors by the LASSO regression model based on 99 patients in the training cohort, where the optimal log(λ) value of −3.66 was chosen according to 8-fold cross validation (Figures 3A,B). These features were displayed as an equation in Supplementary Files.

The radiomics model incorporated above 12 radiomics features, yielding a KS value of 0.519, AUC 0.829 (95% CI: 0.750–0.908), in the training cohort and KS value of 0.423, AUC 0.727 (95% CI: 0.570–0.884), in the testing cohort (Figures 3C–F). The sensitivity, specificity, positive predictive value (PPV), negative predictive value (NPV), and accuracy of the model in the two cohorts are presented in Supplementary Table 2. The GiViTI calibration belt revealed no significant deviations by the GiViTI calibration test in both cohorts (*P* = 0.430, *P* = 0.986) (Figures 3G,H).

**Figure 4:**
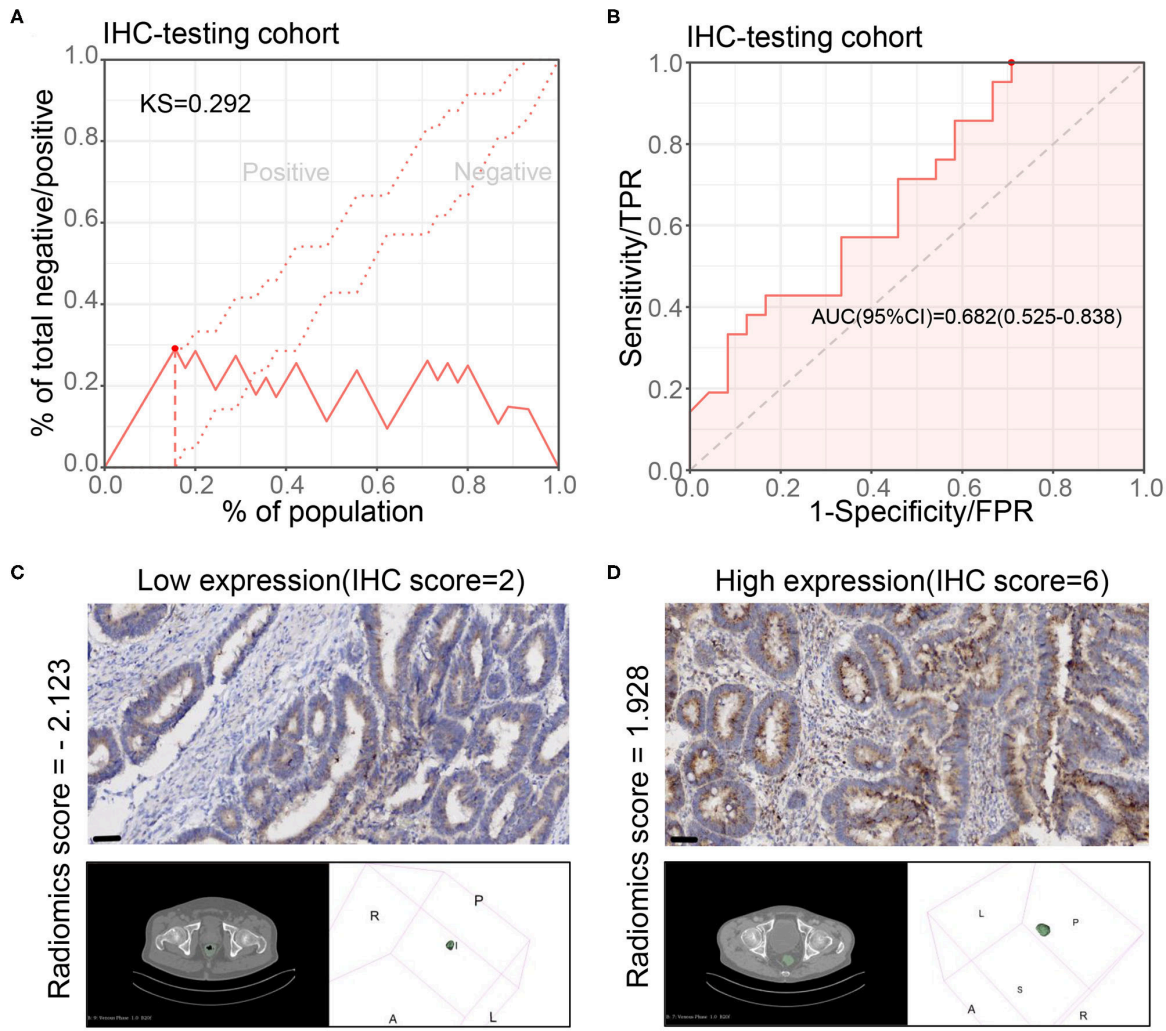
Performance of radiomics model for *CXCL8* assessment in the immunohistochemical testing cohort. **(A,B)** Kolmogorov–Smirnov (KS) curve and receiver operating characteristic (ROC) curve of the radiomics model for assessing the *CXCL8* expression in the immunohistochemical testing cohort (*N* = 45). KS > 0.2 means that the model has a good prediction accuracy. **(C,D)** Two typical cases of patients with high and low *CXCL8* in tumor tissues by immunohistochemistry and their radiomics score according to the radiomics model.

We further verified the results in the IHC testing cohort with IHC staining of *CXCL8*. The radiomics model also showed a good performance in this cohort (KS value = 0.292, AUC = 0.682, 95% CI: 0.525–0.838) (Figures 4A,B). The sensitivity, specificity, PPV, NPV, and accuracy are shown in Supplementary Table 2. Two examples displayed that the model prediction results were in good agreement with the IHC results (Figures 4C,D). These results indicated that parts of the radiomics features indeed associated with *CXCL8* expression. The radiomics characteristics reflected by tumor morphology could be influenced by gene expression in the tumors.

### Prognosis Prediction With Combined Radiomics Model in Colorectal Cancer

To further investigate the possibility of radiomics features in clinical application, we performed above *CXCL8*-derived radiomics model for prognostic status prediction of CRC patients in a prognostic testing cohort with 163 patients. As demonstrated in Supplementary Figure 2, the radiomics model had certain ability in prognostic prediction (KS value = 0.308, AUC = 0.641, 95% CI: 0.527–0.756). Patients were predicted as low risk or high risk by the radiomics model. Kaplan–Meier survival analysis revealed a better prognosis in the low-risk group (Log-rank *P* < 0.0001) (Supplementary Figure 2).

**Figure 5:**
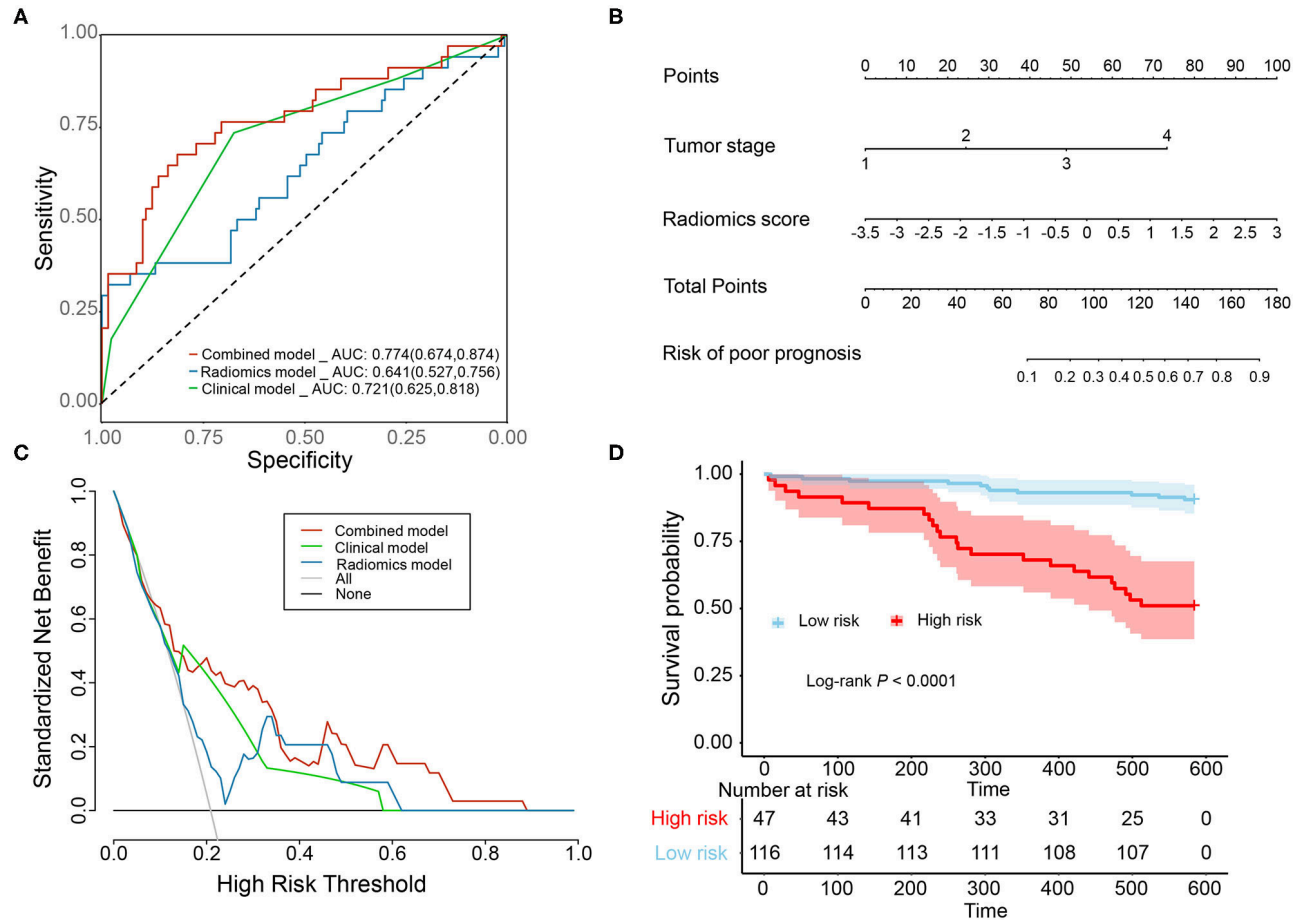
Performance of the combined radiomics model for predicting prognosis in the prognostic testing cohort. **(A)** Nomogram of the *CXCL8*-derived combined radiomics model for prognosis prediction. **(B)** Receiver operating characteristic (ROC) curve comparison of the clinical model, *CXCL8*-derived radiomics model, and *CXCL8*-derived combined radiomics model for prognosis prediction in the prognostic testing cohort (*N* = 163). **(C)** The decision curve analysis for the above three models. **(D)** Kaplan–Meier analysis of the overall survival probability in CRC patients stratified by the *CXCL8*-derived combined radiomics model (High risk vs. Low risk). CRC, colorectal cancer.

Furthermore, we analyzed the combination of clinical tumor stage data and radiomics model and observed that the combined radiomics model (combined tumor stage data and radiomics model) results in better performances in prognosis prediction (AUC = 0.774, 95% CI: 0.674–0.874) than the clinical model (AUC = 0.721, 95% CI: 0.625–0.818) and radiomics model (Figure 5A and Supplementary Table 2). The combined radiomics model that incorporated the clinical tumor stage data and radiomics score is presented as the nomogram (Figure 5B). The decision curve analyses based on the clinical model, radiomics model, and combined radiomics model are shown in Figure 5C. The analysis revealed that the combined radiomics model had a higher net benefit than other schemes. Hence, we tend to choose the combined radiomics model in clinical utility.

In the prognostic testing cohort, patients were stratified into high-risk and low-risk patients based on the cutoff value of combined radiomics model. As shown in Figure 5D, Kaplan–Meier survival analysis demonstrated a significant poor OS (Log-rank *P* < 0.0001) in predicted high-risk patients, which suggested that the predictive value in prognosis of the *CXCL8*-derived combined radiomics model was reliable and practical.

## Discussion

Herein, we firstly identified *CXCL8* as a hub gene related to prognosis in cancers. In two clinical study cohorts, patients with high *CXCL8* levels showed poor prognosis. The underlying mechanisms of different *CXCL8* levels in CRC involved neutrophil chemotaxis and leukocyte migration regulation based on RNA-seq analysis in 95 CRC patients. Subsequently, 12 radiomics features were screened by LASSO method for *CXCL8* expression assessment in 99 CRC patients, and the novel model incorporating above 12 features showed good performance in training, testing, and IHC testing cohorts with AUC of 0.829, 0.727, and 0.682, respectively. We further evaluated whether the *CXCL8*-derived radiomics model had the ability of prognosis prediction in CRC patients. Our results found that the radiomics model could predict the prognosis of patients. Notably, the radiomics model with integrated tumor stage data has a better prognostic ability. Additionally, significant differences were found in survival probability between the high-risk and the low-risk patients based on the combined radiomics model.

*CXCL8* has been shown to promote tumor development in humans in a variety of ways. Recent studies have suggested that this cytokine can significantly affect the accumulation of immunosuppressive and tumor-promoting immune cells by interfering with the infiltration of leukocytes into tumors (24, 25). Tumor-derived *CXCL8* signaling can bias the tumor microenvironment toward immunosuppression through the transport of neutrophils and myeloid inhibitory cells [myeloid-derived suppressor cells (MDSCs)] with local resistance to antitumor immune responses (24, 26, 27). Large randomized studies revealed that elevated serum or plasma *CXCL8* was correlated with reduced clinical benefit of immune-checkpoint inhibitors (13, 14). Interestingly, we firstly used radiomics features to approach the evaluation of *CXCL8* expression levels, then applied the developed *CXCL8*-derived radiomics model to predict patients' prognoses in an independent testing cohort. The results demonstrated that high *CXCL8* in tumor tissues was positively associated with poor prognosis, which is in line with the above studies. Transcriptome sequencing suggested that the underlying mechanism was through regulating immune- and inflammatory-related pathways, while the basic and animal experiments are needed to verify results in the future.

Radiomics can perform quantitative analysis of lesions through a large number of radiological characteristics, which effectively solved the problem that tumor heterogeneity is difficult to be quantitatively evaluated. This has an important clinical application value and has been widely used to predict the prognosis of various cancer patients and improve the treatment strategies (19, 28, 29). Besides, previous studies have proposed that gene expression and pathway status could be evaluated by radiomics features in lung cancer and glioblastoma (30, 31). To our knowledge, no study has explored the underlying connections of imaging features and *CXCL8* expression in CRC. In this study, we identified and validated 12 imaging features with *CXCL8* levels. Due to the significant association between *CXCL8* and OS, we performed the *CXCL8*-derived radiomics model to predict the prognosis of CRC patients in a cohort with 163 patients. ROC analysis, decision curve, and Kaplan–Meier survival analysis demonstrated that the model had ability in poor prognosis prediction. Multiple perspectives data of tumors such as genomics, transcriptomics, metabonomics, clinical features, and radiomics have been combined to systematically describe the tumors. Clinically, the prognosis of cancers is closely related to the stage of tumors. Hence, in this study, we further compared the performance of clinical model, radiomics model, and combined radiomics model in prognosis prediction. The results revealed that the combined radiomics model had a higher AUC (0.774) compared with radiomics model (0.641) and clinical model (0.721). The decision curve demonstrated that the combined radiomics model achieved the best net benefit. Considering the effectiveness of clinical application, we finally recommend the combined radiomics model.

Although the *CXCL8*-derived combined model has been successfully applied in CRC prognosis prediction in our study, it had some limitations that need to be acknowledged. Firstly, this study is a hospital-based single-center retrospective analysis, in which the genes and clinical characteristics of patients may not be representative of the population. Although we randomly divided the participants into independent training and testing cohort at a 7:3 ratio, there was still a lack of generalization and robust evaluation of the model. Secondly, some clinical studies have shown that *CXCL8* disorder is related to tumor prognosis. However, the specific mechanism of *CXCL8* affecting tumor prognosis needs further investigation in cells and animals. Thirdly, the region of interest of the tumor is drawn manually by experienced professionals, which is time-consuming and may increase the variability between observers. In the future, our results should be verified in a prospective study of patients from multiple centers using more efficient and accurate lesion segmentation algorithms.

## Conclusions

Our study demonstrated that radiomics features could perform *CXCL8* assessment and prognosis prediction in CRC patients, which has a positive application value for guiding clinical decision by analyzing preoperative CT images.

## Data Availability Statement

The original contributions presented in the study are publicly available. This data can be found here: https://bigd.big.ac.cn/gsa-human/browse/HRA000235.

## Ethics Statement

The studies involving human participants were reviewed and approved by the medical ethics review committee of Dazhou Central Hospital. The patients/participants provided their written informed consent to participate in this study.

## Author Contributions

FZ, JC, and YC participated in the study design and manuscript preparation. JL and JZ performed RNA-seq data analysis and prepared figures. ZZ and QL performed data analysis. BH enrolled the patients. YeZ revised the manuscript. All authors contributed to the article and approved the submitted version.

## Conflict of Interest

The authors declare that the research was conducted in the absence of any commercial or financial relationships that could be construed as a potential conflict of interest.
