# Supplementary material for: A Novel Model Based on CXCL8-Derived Radiomics for Prognosis Prediction in Colorectal Cancer
Source: Front Oncol. 2020 Oct 14;10:575422. doi: 10.3389/fonc.2020.575422 (PMC7592598; doi:10.3389/fonc.2020.575422)
Supplement: Supplementary file 1 [file Data_Sheet_2.pdf]

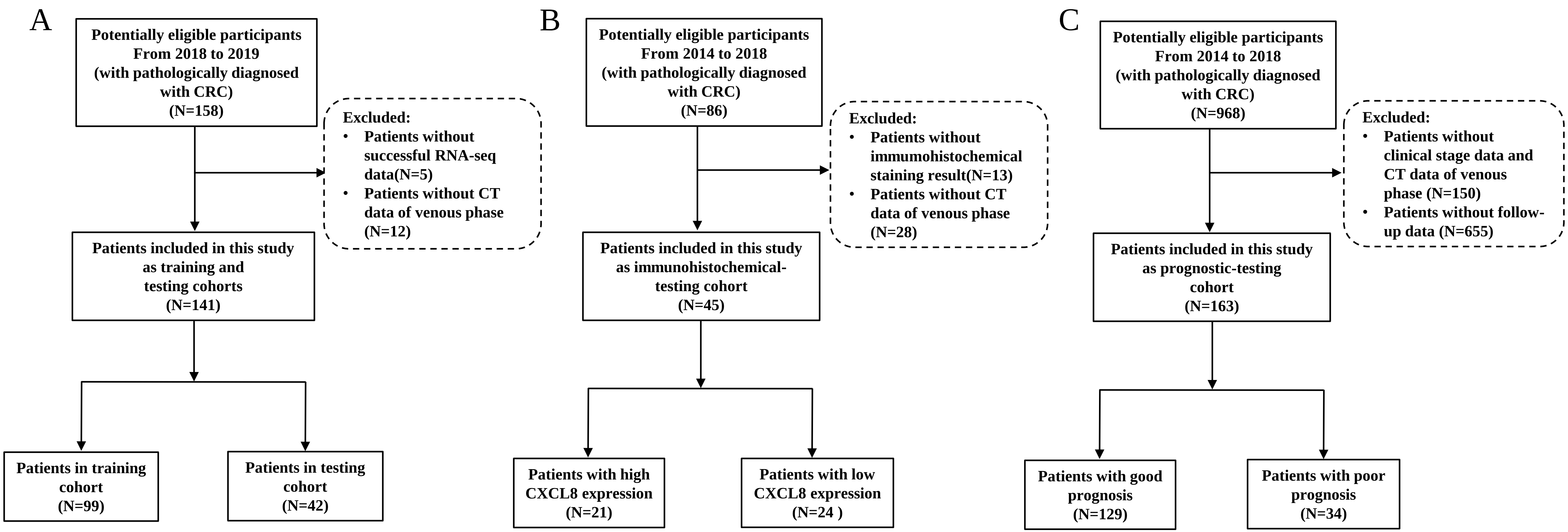

Supplementary Figure 1. Patients' inclusion and exclusion criteria of training cohort, testing cohort, immunohistochemical-testing cohort, and prognostic-testing cohort.

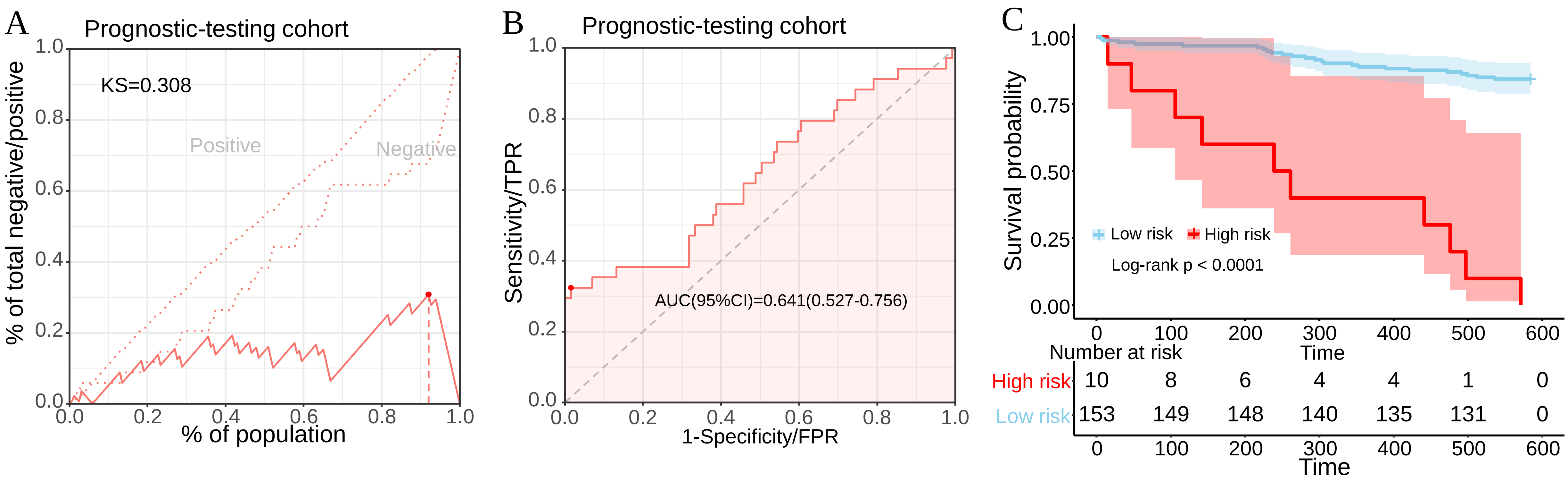

Supplementary Figure 2. Performance of the radiomics model for prognosis prediction in prognostic-testing cohort. (A-B) Kolmogorov-Smirnov (KS) curve and ROC curve of radiomics model for prognosis prediction in prognostic-testing cohort (N=163). (C) Kaplan–Meier analysis of the overall survival probability in CRC patients stratified by *CXCL8* derived radiomics model (High risk *versus* Low risk).
