# Supplementary material for: A Novel Model Based on CXCL8-Derived Radiomics for Prognosis Prediction in Colorectal Cancer
Source: Front Oncol. 2020 Oct 14;10:575422. doi: 10.3389/fonc.2020.575422 (PMC7592598; doi:10.3389/fonc.2020.575422)
Supplement: Supplementary file 2 [file Data_Sheet_1.docx]

Supplementary Table1. Characteristics of Patients in training cohort and testing cohort.

| **Characteristic** | **Training cohort (N=99)** | |  | **Testing cohort (N=42)** | |  | ***P*** |
| --- | --- | --- | --- | --- | --- | --- | --- |
|  | **High (n=54)** | **Low (n=45)** | ***P*** | **High (n=20)** | **Low (n=22)** | ***P*** |  |
| Age (mean ± SD) | 59.09±12.95 | 61.42±11.42 | 0.491 | 59.68±10.85 | 62.45±11.463 | 0.427 | 0.825 |
| Sex, No. (%) |  |  | 0.627 |  |  | 0.491 | 0.994 |
| Male | 31 (0.57) | 28 (0.62) |  | 13 (0.65) | 12 (0.55) |  |  |
| Female | 23 (0.44) | 17 (0.38) |  | 7 (0.35) | 10 (0.45) |  |  |
| Tumor stage, No. (%) |  |  | 0.950 |  |  | 0.016 | 0.988 |
| 0 | 2 (0.04) | 1 (0.02) |  | 0 (0.00) | 1 (0.05) |  |  |
| I | 13 (0.24) | 12 (0.27) |  | 4 (0.20) | 7 (0.32) |  |  |
| II | 15 (0.28) | 12 (0.27) |  | 11 (0.55) | 2 (0.09) |  |  |
| III | 21 (0.39) | 16 (0.36) |  | 5 (0.25) | 9 (0.41) |  |  |
| IV | 3 (0.06) | 4 (0.09) |  | 0 (0.00) | 3 (0.14) |  |  |
| Tumor sites, No. (%) |  |  | 0.431 |  |  | 0.513 | 0.793 |
| Rectum | 34 (0.63) | 34 (0.76) |  | 14 (0.70) | 14 (0.64) |  |  |
| Right colon | 11 (0.20) | 4 (0.09) |  | 4 (0.20) | 3 (0.14) |  |  |
| Left colon | 8 (0.15) | 6 (0.13) |  | 2 (0.10) | 5 (0.23) |  |  |
| Multiple tumors | 1 (0.02) | 1 (0.02) |  | 0 (0.00) | 0 (0.00) |  |  |

The *P* value of age: Mann-Whitney U. The *P* value of sex, tumor stage, tumor sites: chi-square test.

Supplementary table 2. Performance of the models in different cohorts.

| **Items** | **Radiomics model** | | | | **Clinical model** | **Combined model** |
| --- | --- | --- | --- | --- | --- | --- |
|  | **Training cohort** | **Testing cohort** | **IHC-testing cohort** | **PI-testing cohort** | **PI-testing cohort** | **PI-testing cohort** |
| AUC | 0.829(0.750-0.908) | 0.727(0.570-0.884) | 0.682(0.525-0.838) | 0.641(0.527-0.756) | 0.721(0.625-0.818) | 0.774(0.674-0.874) |
| Specificity | 0.756 (0.467-0.933) | 0.727(0.364-1.000) | 0.583(0.250-1.000) | 0.984(0.395-1.000) | 0.674(0.597-0.752) | 0.829(0.667-0.922) |
| Sensitivity | 0.796(0.611-1.000) | 0.800(0.400-1.000) | 0.857(0.333-1.000) | 0.382(0.206-0.882) | 0.735(0.588-0.882) | 0.706(0.529-0.882) |
| NPV | 0.769(0.637-1.000) | 0.783(0.630-1.000) | 0.818(0.615-1.000) | 0.854(0.825-0.935) | 0.906(0.857-0.954) | 0.915(0.872-0.959) |
| PPV | 0.796(0.688-0.914) | 0.722(0.576-1.000) | 0.636(0.526-1.000) | 0.857(0.271-1.000) | 0.373(0.305-0.455) | 0.511(0.379-0.689) |
| Accuracy | 0.778(0.707-0.848) | 0.738(0.619-0.857) | 0.689(0.578-0.800) | 0.847(0.497-0.883) | 0.687(0.613-0.755) | 0.798(0.693-0.871) |

Abbreviation: IHC-testing cohort, Immunohistochemistry testing cohort; PI-testing cohort, prognosis independent testing cohort; AUC, Aera under curve; PPV, positive predictive value; NPV, negative predictive value.
