# Supplementary material for: A Novel Model Based on CXCL8-Derived Radiomics for Prognosis Prediction in Colorectal Cancer
Source: Front Oncol. 2020 Oct 14;10:575422. doi: 10.3389/fonc.2020.575422 (PMC7592598; doi:10.3389/fonc.2020.575422)
Supplement: Supplementary file 3 [file Data_Sheet_3.docx]

**Supplementary Files**

Radiomics score =

-3.413+3.680*original_shape_Elongation+2.885*wavelet_HHH_glcm_ClusterProminence+0.00000656*wavelet_HHH_ngtdm_Busyness+0.0552*wavelet_HHL_glrlm_LongRunLowGrayLevelEmphasis-410.813*wavelet_HHL_ngtdm_Strength-0.000380*wavelet_HLL_firstorder_Kurtosis+0.00157*wavelet_HLL_glcm_ClusterProminence-2.357*wavelet_HLL_glcm_MCC+31.633*wavelet_HLL_glszm_SmallAreaLowGrayLevelEmphasis+0.00268*wavelet_LHL_glszm_SmallAreaHighGrayLevelEmphasis+0.00527*wavelet_LLL_firstorder_Median-0.00472*wavelet_HHL_firstorder_Kurtosis
